# Surgical Decision-Making in Thyroiditis: A Review Article

**DOI:** 10.7759/cureus.46055

**Published:** 2023-09-27

**Authors:** Sahar M Alnefaie

**Affiliations:** 1 Breast and Endocrine Surgery, Taif University, Taif, SAU

**Keywords:** de quervain thyroiditis, acute thyroiditis, operation, hashimoto’s thyroiditis, surgery, thyroiditis

## Abstract

Thyroiditis is a common thyroid disorder worldwide. Its commonest variant is Hashimoto’s thyroiditis (HT). The usual presenting symptom is hypothyroidism at the time of diagnosis. Most patients are asymptomatic. A few cases may experience more obvious symptoms. The standard treatment is medical, and surgery is rarely indicated. This review aims to shed light on the most relevant articles discussing surgery in thyroiditis and compare them. The author included 16 relevant articles focused on the surgical management of thyroiditis. Of the studies, 10 were designed as retrospective reviews, four as prospective studies, and two as systematic reviews. The included studies aimed to focus on certain objectives. Three of them discussed indications of surgical intervention in cases of thyroiditis. Five covered postoperative complications. Six looked at the thyroid cancer association with thyroiditis. Two explored the impact of thyroiditis on surgery. Five discussed the postoperative improvement of symptoms and the effect on the quality of life. All the studies listed in this review engaged cases of HT except two. This review concentrates on conceptualizing and organizing the parameters in the minds of surgeons who manage cases of thyroiditis to enable them to decide whether to operate or not. These factors are indications of surgery, improvement of local compressor symptoms post thyroidectomy like dysphasia and shortness of breath, risk of thyroid cancer, intraoperative difficulty, and postoperative complications. Different variants of thyroiditis cases are treated medically, and surgery is rarely indicated. The surgeons should consider the mentioned factors affecting their decision to operate on this special entity. Thus, each case should be individualized in deciding whether to operate or not by assessing the benefit-risk ratio.

## Introduction and background

Thyroiditis is defined as an inflammatory disorder of the thyroid gland [1]. It is a common thyroid disorder affecting many individuals worldwide. The prevalence of HT differs based on geographic positions, ranging from 5.8% to 14.2% [2]. It is classified into two categories: autoimmune thyroiditis, which includes HT and Riedel thyroiditis, and infected thyroiditis. The commonest variant is HT. The usual presenting symptom is hypothyroidism, which usually improves with thyroxin replacement. Otherwise, the majority of HT patients are asymptomatic. A few cases may experience neck pain, neck tightness, diffuse goiter, or even more obvious symptoms, such as dysphagia, shortness of breath, and solitary or multinodular goiter that may harbor malignancy.

The standard treatment is medical in the form of replacing thyroid hormone in cases of hypothyroidism. Acute suppurative thyroiditis is managed with antibiotics. Corticosteroids are prescribed for some cases of Riedel thyroiditis. Surgery is uncommonly indicated in thyroiditis. The most frequent indications are abscess for drainage, compressive symptoms, and the presence or suspicion of malignancy. However, surgeries in cases of thyroiditis carry the risk of intraoperative and postoperative complications. Therefore, the clinical literature review revealed that some surgeons are reluctant to operate on these cases if clinically indicated while others have a low threshold for operations in such cases.

In this review, the author aims to shed light on the most relevant articles that discussed surgery for different types of thyroiditis and compare them, highlighting their rationale for operating on these cases or treating them with medical treatment, and the consequences of their decisions. The data reported by different authors regarding the management plan of thyroiditis are diverse. Thus, the author comes up with a conceptual framework from her point of view, based on her clinical experience in this field. This will hopefully help surgeons in deciding whether to operate on thyroiditis cases or not.

## Review

Methodology

**Figure 1:**
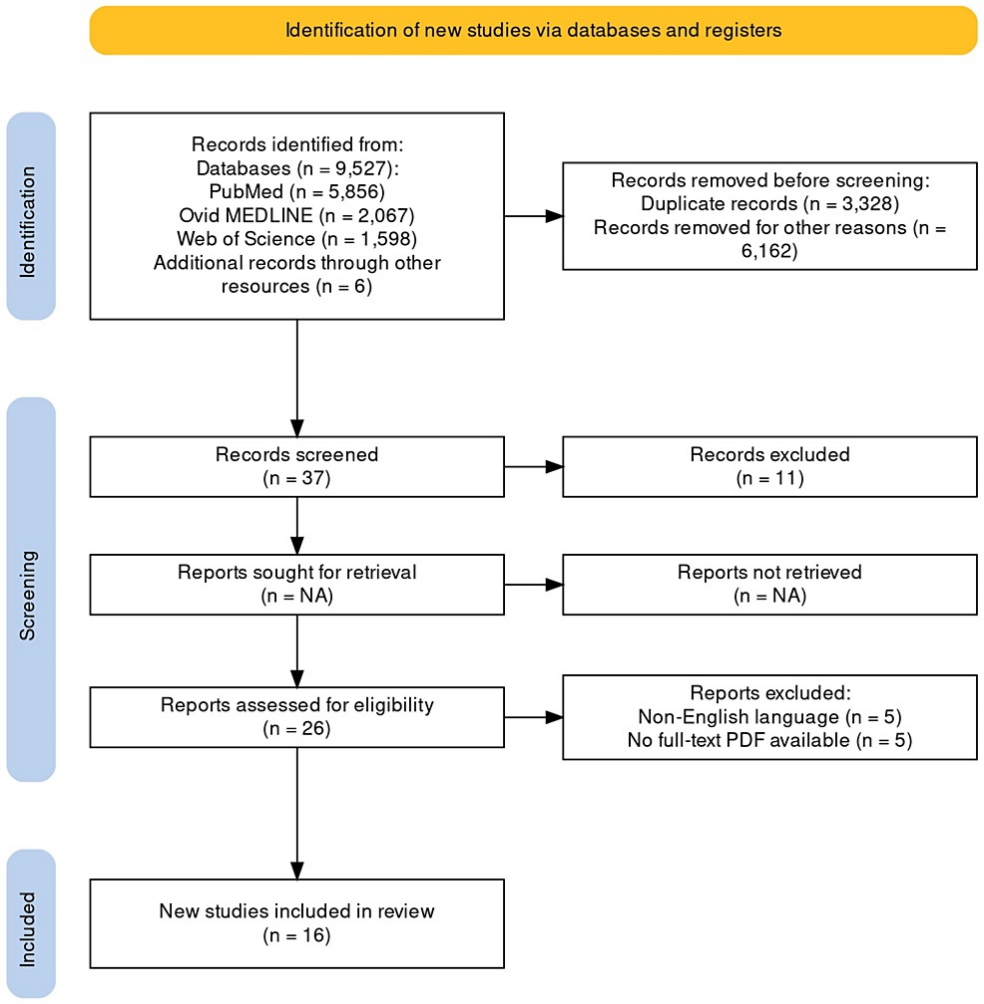
PRISMA 2020 flow diagram. The PRISMA 2020 statement: an updated guideline for reporting systematic reviews [3]. PRISMA, Preferred Reporting Items for Systematic Reviews and Meta-Analyses; NA, not applicable; N, number

Web of Science, PubMed, Ovid MEDLINE, and other electronic databases were used to search the English-language literature. The MeSH terms used are surgery for thyroiditis, operation for thyroiditis, nonmedical treatment of thyroiditis, thyroidectomy for thyroiditis, surgery for HT, acute/subacute thyroiditis, de Quervain thyroiditis, and Riedel thyroiditis. The author’s experience in the subject area helped the preservation of related publications. The 16 articles in this review meet the following inclusion criteria: They are all full-text studies, deemed significant in the field of thyroiditis surgery, and encompass all available years. Non-English articles and case reports were excluded, as well as articles with unavailable full-text PDFs. The Preferred Reporting Items for Systematic Reviews and Meta-Analyses (PRISMA) research approach is displayed in Figure 1.

A summary of the main characteristics of the 16 included studies is reported in Table 1. This includes descriptive findings such as author names, publication year, country, methods, sample size, objectives, outcome, thyroiditis type, surgery indication, operation type, and postoperative complications.

**Table 1:** Characteristics of the included studies. HT, Hashimoto’s thyroiditis; DTC, differentiated thyroid cancer; RLN, recurrent laryngeal nerve; MNG, multinodular goiter; TT total thyroidectomy; GD, Graves’ disease; ATD, autoimmune thyroid disease; AST, acute suppurative thyroiditis; IJV, internal jugular vein; QoL, quality of life; anti-TPO-Ab, anti–thyroid peroxidase antibody; BG, benign goiter; NA, not available

Authors’ names	Year/ country	Methods/Sample size	Objectives	Outcome	Type of thyroiditis	Indication of surgery	Operation performed	Postoperative complications
Pisanu et al. [4]	2003/Italy	Retrospective review of cl. Records, Cohort study of 44 HT (33 DTC, 11 no DTC)	Indications for thyroidectomy in HT, the frequency of coexistence of HT and DTC, and the impact of HT on the management of DTC	An adequate follow-up of patients with HT may permit an early diagnosis of DTC and its appropriate management.	HT	As per the result of FNAC	TT	Morbidity rate was not increased very much by HT
Ravikumar et al. [5]	2018/India	Retrospective review of 724 patients (A non-thyroiditis 452, B thyroiditis 272)	Incidence of both transient and permanent complications in patients undergoing TT in goiters associated with thyroiditis	Incidences of transient complications are higher in thyroiditis patients than non-thyroiditis patients while the permanent complications are comparable between them.	HT + nodular goiter with associated thyroiditis	Compressive symptoms (dysphagia, choking sensation, or voice change), suspicious or proven malignancy, retrosternal extension, toxic MNG, and cosmesis	TT	The incidence of temporary hypocalcemia and transient RLN palsy is higher in thyroiditis cases.
Saadi et al. [6]	2019/United States	Retrospective review of 62 thyroiditis	Thyroid characteristic, degree of operative difficulty, and postoperative outcomes in HT and GD compared to patients without ATD	HT and GD undergoing TT have significantly increased qualitative changes of the thyroid that correlate with increased surgical difficulty and length of hospital stay.	HT and GD	Symptomatic cases	Total/subtotal thyroidectomy	Neck hematoma, vocal cord paralysis, hypocalcemia, parathyroid auto-transplantation, and surgical site infection
Bage and Kalatharan [7]	2021/India	Prospective, hospital-based descriptive study of 30 patients	Identifying improvement of pressure symptoms, pain in the thyroid gland, chance of malignancy, and cosmetic after TT.	Total relief of compressive and painful symptoms and low incidence of/nil major complications	HT	Symptomatic HT (pain, tenderness and pressure symptoms)	TT	Transient hypocalcemia, RLN palsy, and pain
Lafontaine et al. [8]	2021/Australia	A systematic review of 200 cases of AST described in 148 articles	To update understanding of this medical emergency	Complications and sequelae of AST are numerous, whether from the disease process itself or from its management.	AST	Failed needle aspiration and medical treatment (antibiotics or antifungal) and presence of abscess and/or fistula	Incision and drainage, partial thyroidectomy, and TT	IJV thrombophlebitis, mediastinitis, pericarditis, esophageal perforation, fistula, obstruction, severe laryngeal edema, Horner's syndrome, multisystem organ failure, and death
Peterson [9]	1957/United States	Retrospective study of 757 patients	To ascertain the relationship of carcinoma of the thyroid gland to both forms of lymphocytic thyroiditis	(1) The various microscopic variants of lymphocytic thyroiditis belong to a single disease. (2) No increased incidence of cancer	HT	Nodular goiter, pressure symptoms, and suspicion of malignancy	Lobectomy, subtotal thyroidectomy, or resection of isthmus only	NA
Lindem and Clark [10]	1969/Utah, United States	Retrospective study of 46 patients	To identify indications for thyroidectomy in HT and Riedel’s thyroiditis	Indications are the presence of a mass to be differentiated from carcinoma and local pressure symptoms, hyperthyroidism, cosmetic deformity, and lack of response to medical therapy	HT and Riedel’s thyroiditis	Presence of a mass to be differentiated from carcinoma and pressure symptoms, hyperthyroidism, cosmetic deformity, and lack of response to medical therapy	Near-total thyroidectomy	Wound seroma, transient hypocalcemia, and transient RLN paralysis
Clark et al. [11]	1980/United States	Retrospective study of 75 patients	(1) What conditions coexist with HT. (2) To select patients with HT who are at high or low risk for thyroid cancer	Patients with HT can be separated into those at low risk and those at high risk for cancer.	HT	Nodular thyroid gland, diffusely enlarged gland, and solitary nodule	Open biopsy, lobectomy, sub/near TT, and/or neck dissection	Transient hypocalcemia, vocal cord palsy, and corneal abrasion
McManus et al. [12]	2011/United States	Retrospective study of 133 patients with symptomatic HT	Patients with symptomatic HT were evaluated for improvement of preoperative symptoms after thyroidectomy.	Majority of HT patients with significant symptoms appear to benefit from thyroidectomy.	HT	Compressive symptoms, suspicion of malignancy, or a goiter that is increasing in size	Lobectomy, subtotal, or TT	Transient or permanent complications
Pradeep et al. [13]	2011/India	Retrospective study of 271 patients	(1) To compare the indications, complications, and cancers in patients operated for HT with other BG. (2) The effect of surgery on minor discomfort symptoms	Discomfort in swallowing and tightness in the neck are relieved after surgery. Thyroidectomy is safe and has a low permanent complication. There was no associated malignancy.	HT	Suspicion of malignancy, tracheal/esophageal compression, pain, cosmetic, discomfort in swallowing without obvious dysphagia, and sensation of tightness in neck	TT	No case of permanent hypocalcemia/RLN palsy but temporary hypocalcemia
Seifman et al. [14]	2011/Australia	Retrospective study of 112 patients	To document the results of cytology and the rate of malignancy in HT	Due to increased rate of malignancy and diagnostic uncertainty with HT, we recommend routine preoperative thyroid antibody.	HT	Diagnosis with malignancy, risk of malignancy, pressure symptoms, thyrotoxicosis, and patient request	TT	Permanent RLN palsy, hypoparathyroidism, and postoperative hemorrhage
Promberger et al. [15]	2014/Austria	A prospective cohort study of 248 patients	To evaluate (1) QoL-related outcome and (2) the change in symptom load in the long-term after TT	Patients with HT might benefit in terms of QoL. It should not be recommended for patients with elevated anti-TPO-Ab.	HT	Symptomatic HT, like dysphagia and tightness in the neck	Lobectomy, total or near-total thyroidectomy	Transient or permanent hypocalcemia or RLN injury, postoperative hemorrhage, and wound infection
Zivaljevic et al. [16]	2015/Serbia	Prospective cohort study of 143 patients	To compare the health and QoL of HT and BG patients before and after TT.	The QoL of HT and BG patients is impaired, and it is improved after thyroidectomy	HT	Toxic adenoma, nodular goiter, MNG, HT, and suspicious malignancy	Hemithyroidectomy and TT	Transient and permanent complications
Ranganath et al. [17]	2016/United States	Retrospective study of 10 patients	To review our experience with de Quervain thyroiditis	Rarely treated surgically. If surgery is indicated, it will be risky.	de Quervain	Dysphagia, nodules with atypia or suspicious for cancer	Lobectomy, TT, and lymph node sampling	NA
Gan et al. [18]	2021/China	A systematic review and meta-analysis of nine studies	To investigate the significance of HT for the postoperative complications	HT only increased the risk of the transient hypocalcemia compared to BG.	HT	NA	Lobectomy and TT	Transient, permanent hypocalcemia, or RLN palsy
Thomusch et al. [19]	2018/Germany	A prospective multicenter European study 18,955 (2,488 HT and 16,467 MNG)	Incidence of thyroid surgery-specific complications after operations for ATD	Surgery for ATD is safe in comparison with MNG in terms of general complications and RLN palsy. Yet, increased risk of postoperative hypoparathyroidism	HT, GD, de Quervain thyroiditis, and Riedel thyroiditis	-Suspicious or malignant thyroid, -hyperthyroidism refractory to medication, compressive symptoms, disease recurrence, and GD with ophthalmopathy	Lobectomy, sub/near TT and TT	Temporary and permanent hypocalcemia and RLN palsy, wound infection, pulmonary, cardiovascular, thromboembolic, and bleeding

Results

In this review, the author included 16 relevant articles focusing on the surgical management of thyroiditis. Of these, three were published before 2000. The locations of the publishing authors demonstrated good variation, including the United States, Europe, Asia, and Australia. Of these studies, 10 were designed as retrospective reviews [4-6,9-14,17], four as prospective studies [7,15,16,19], and two as systematic reviews [8,18]. The sample size ranged from 10 to 18,955 patients.

The included studies aimed to focus on certain objectives. Three of them discussed indications of surgical intervention in cases of thyroiditis [4,10,13]. The others included indications of surgery in thyroiditis although it was not the main objective of their studies. The reported surgical indications are mainly compressive symptoms such as dysphagia, choking sensation, or voice change, as well as pain, cosmetic deformity, retrosternal extension, toxic multinodular goiter (MNG), no response to medical therapy, and more seriously, presence or suspicion of malignancy in the thyroid gland [4,5,10,13]. In cases of acute suppurative thyroiditis (AST), surgery is indicated for an abscess and/or fistula [8].

Six studies looked at the thyroid cancer association with thyroiditis [4,7,9,11,13,14]. Some studies reported an increased risk of cancer [4,14], while others did not [9,13]. One study categorizes patients into high and low risk for malignancy. Patients with diffuse or nodular goiter are at low risk of carcinoma, whereas those with a single or prominent nonfunctioning or cold thyroid nodule on radioiodine scan are at high risk of thyroid carcinoma [11]. One study concluded that “in longstanding cases of HT, the risk of malignant transformation is prevented due to total thyroidectomy” [7].

Postoperative improvement of local symptoms and quality of life (QoL) were discussed in five studies [7,12,13,15,16]. All these studies have shown that patients with thyroiditis had significantly improved preoperative symptoms after thyroidectomy and benefitted regarding QoL. With more elaboration, there was a complete improvement in compressive and painful symptoms [7]. Most symptomatic HT patients had relief of their symptoms after surgery [12]. Patients with discomfort in deglutition and tight neck are relieved post-thyroidectomy [13]. HT cases may benefit in terms of QoL, specifically, physical, emotional, mental, and social functioning. The QoL, with domains of physical, mental, and sexual life, of both HT and benign goiter patients is diminished and significantly improves postoperatively [16].

Two studies deal with the impact of thyroiditis on surgery [4,6]. Saadi et al. concluded that HT and Graves’ disease undergoing total thyroidectomy have significantly qualitative changes of the thyroid gland, which correlate with increased surgical difficulty and lengthy hospitalization [6]. Another issue is intraoperative differentiation between chronic cervical lymph nodes in level VI in cases of HT and metastatic lymph nodes from coexisting thyroid cancer or even a parathyroid gland in inexperienced hands [4].

Five studies concerned postoperative complications [5,6,13,18,19]. The incidence of temporary hypocalcemia and transient recurrent laryngeal nerve (RLN) palsy was found to be higher in thyroiditis cases [5]. Similar findings in three other studies showed a low incidence of permanent hypocalcemia and RLN palsy, but temporary hypocalcemia is more common [13,18,19]. The rate of transient hypocalcemia was significantly higher in the HT group (16.9%) compared with the benign nodule (BN) group (13.2%; *P *< 0.001). However, there was no significant difference in the risk for permanent hypoparathyroidism between the two groups (1.17% and 0.99%, *P *= 0.431) [18]. Moreover, there were no significant differences between the HT group and the BN group in the risk for transient RLN palsy (3.93% and 3.91%; *P *= 0.842) and in the risk for permanent RLN palsy (1.15% and 0.78%; *P *= 0.234) [18].

All the studies listed in this review engaged cases of HT except two [8,17]. Two studies reviewed or updated the uncommon types of thyroiditis, such as AST and de Quervain thyroiditis [8,17]. They concluded that the consequences of AST are numerous, either from the disease process itself or from its treatment [8]. de Quervain thyroiditis is rarely managed surgically, and if surgery is indicated, it carries a risk of complications [17]. The extent of surgical intervention was determined as per the indication of surgery. It ranged from thyroid abscess drainage, lobectomy, sub- or near-total thyroidectomy, and total thyroidectomy to neck dissection.

Discussion

Thyroiditis is a common disease worldwide, especially HT. Most of the cases are treated with medications; however, a few require surgical treatment. Many reports in the literature cover the medical management of this disease, yet few of them discuss the surgical part in the form of surgery indications, surgery type, and postoperative complications, in comparison to non-thyroiditis cases. This is the first review, to the best of the author’s knowledge, that gathers, reviews, and thoroughly and deeply compares the previously published reports that discussed the different aspects of surgery in thyroiditis.

**Figure 2:**
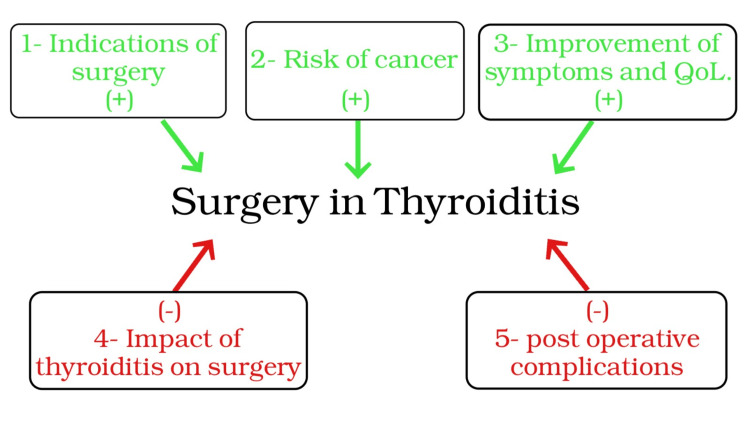
Conceptual framework of factors playing a role in surgery decisions in thyroiditis. Image credit: Sahar M. Alnefai. QoL, quality of life

This review concentrates on conceptualizing and organizing the parameters to guide surgeons who manage cases of thyroiditis in deciding whether to operate or not. From the author’s point of view, five main factors should be considered in such situations. The author has created a conceptual framework of these five parameters, which are recommended in surgery decisions for thyroiditis, as illustrated in (Figure 2).

The positively influencing factors that encourage the surgeon to operate are discussed next.

The indications for operation include compressive symptoms, namely, dysphagia or shortness of breath with elaboration of its severity and impact on daily activities. Other indications are lack of response to medical therapy, nodular variant of HT, and cosmetic deformity [20]. More seriously, the presence or suspicion of malignancy in the thyroid gland based on fine-needle aspiration cytology is also an indicator.

The risk of cancer in patients with thyroiditis exists mainly in the case of solitary or cold nodule, positive family history of thyroid cancer, and rapidly growing goiter. The prevalence of thyroid cancer in HT in different surgical series ranges from 0.4% [21] to 28% [22]. Two studies reported increased risk [4,14], while two did not [9,13]. Moreover, Abbasgholizadeh et al. published a meta-analysis study, concluding that HT is associated with all types of thyroid malignancies, except follicular and anaplastic thyroid cancer: “Studies with high risk of bias, the high level of heterogeneity between different studies, and the limited number of well-designed prospective studies make the available evidence uncertain” [23]. This shows that there are inconsistent findings regarding the risk of cancer coexistence with thyroiditis, except for the well-known risk of lymphoma in long-standing HT cases. Yet, this risk should be borne in the mind of the surgeon managing thyroiditis cases.

Improvement of Preoperative Local Symptoms and QoL After Thyroidectomy

HT has been shown to be linked with diminished QoL [24,25]. Thyroiditis patients showed significant improvement in preoperative symptoms as well as enhancement of QoL after thyroidectomy [7,12,13,15,16]. It was found that mechanical symptoms, such as pressure symptoms leading to difficulty in swallowing and neck tightness, are relieved after surgery [12,26]. This is an encouraging outcome of thyroidectomy for both patients and surgeons. However, surgical intervention is recommended in real symptomatic cases, such as compression symptoms in which the risks of the thyroid disease are more than those of the operation [15].

On the other hand, factors that may make the surgeon reluctant to operate on thyroiditis cases are discussed next.

Impact of HT on surgery: Thyroidectomy for thyroiditis is technically more difficult than non-thyroiditis cases, requiring more operative time and longer hospitalization [6]. Due to the intraoperative difficulty of thyroidectomy in thyroiditis cases, operating on them is not recommended generally [27]. This difficulty results in a higher risk of postoperative complications. Another issue is intraoperative differentiation between chronic cervical lymph nodes in level VI in cases of HT and metastatic lymph nodes from coexisting thyroid cancer or even a parathyroid gland in inexperienced hands [4]. Therefore, surgery on thyroiditis cases should be performed by a senior, specialized surgeon who has long experience with difficult thyroidectomies and can manage intraoperative challenges, such as bleeding and difficulty in identification of RLN and/or parathyroid gland. This will hopefully reduce postoperative complications.

Finally, postoperative complications, such as transient or permanent postoperative complications, are the usual concerns of surgeons. The risk of temporary or permanent hypocalcemia and/or RLN palsy may occur after any total thyroidectomy. In this review, the incidence of temporary hypocalcemia and transient RLN palsy was found to be higher in thyroiditis patients [5]. Similar findings in three other studies showed a low incidence of permanent hypocalcemia and RLN palsy, but temporary hypocalcemia is more [13,18,19]. Gan et al. published a meta-analysis study, which revealed that the rate of transient hypocalcemia was significantly higher in the HT group (16.9%) compared with the BN group (13.2%; *P *< 0.001). However, there was no significant difference in the risk for permanent hypoparathyroidism between the two groups (1.17% and 0.99%, *P *= 0.431). Moreover, there were no significant differences between the HT group and the BN group in the risk for transient RLN palsy (3.93% and 3.91%; *P *= 0.842) and in the risk for permanent RLN palsy (1.15% and 0.78%; *P* = 0.234) [18].

In the case of associated thyroid cancer, total thyroidectomy with level VI lymph node dissection was found to be related to more risk of hypocalcemia [28,29]. Meticulous intraoperative dissection can decrease the risk of postoperative hypocalcemia [30]. One study found a statistically significant difference regarding permanent hoarseness of voice in HT cases compared with non-HT cases [27]. Other documented post-thyroidectomy complications in cases of thyroiditis are bleeding, neck hematoma, parathyroid auto-transplantation, and surgical site infection. However, the risk is comparable with non-thyroiditis cases except for temporary hypocalcemia and transient RLN palsy, as they are found to be more in thyroiditis cases [5,6,13,18,19]. Even when thyroid cancer patients were eliminated, postoperative complications were found to be higher in HT patients [27].

The decision of surgery depends on the presence or absence of one or more of these five factors. In some cases, the surgeon might be obligated to operate on thyroiditis patients regardless of the consequences. For example, if a patient with thyroiditis has an absolute indication of surgery such as severe compression symptoms due to nodular goiter. In this situation, the surgeon will be required to take a high-risk consent and counsel the patient about possibly higher postoperative complications, which is due to intraoperative difficulty from the impact of thyroiditis on the thyroid gland. The influencing factors for operating on these cases are a clear indication of surgery, postoperative improvement of symptoms, and elimination of the risk of thyroid cancer. To reduce the risk of postoperative complications, the operation should be performed by an expert endocrine or head-and-neck surgeon. The surgeon needs to plan this operation very well with proper preoperative investigations, utilization of intraoperative nerve monitor, and anticipation of longer operative time.

In other situations, operating on thyroiditis cases may have lower priority. In the case of a patient with thyroiditis presenting with diffuse goiter with no compression symptoms or any suspicion of malignancy but insisting on an operation for a cosmetic purpose, the surgeon should think of all previously mentioned factors. This patient has no absolute indication for operation and having no nodule indicates a lesser likelihood of harboring thyroid malignancy. The patient’s thyroiditis makes the operation more challenging due to the impact of thyroiditis on the surgery technique, anticipating longer hospital stay, and greater postoperative complications. The patient may gain one advantage of undergoing an operation, which is the improvement of preoperative symptoms in the form of a cosmetic outcome. However, this patient should bear in mind that the scar look varies from one patient to another and, more importantly, the negative impact of thyroiditis on surgery technique and postoperative complications. This information should be delivered to the patient by the attending surgeon in a counseling meeting.

In short, each case should be individualized regarding the decision to operate or not by a careful assessment of the benefit-risk ratio, bearing in mind the five influencing factors and the need for proper counseling of the patient.

Limitations

Most of the included studies in this review were designed as retrospective studies. However, only four were prospective studies and two were systematic review. This was due unavailability of strongly designed studies in the current literature. Future reviews of surgery for thyroiditis cases are suggested to include more studies designed as systematic reviews and meta-analyses, randomized controlled studies, and prospective cohort studies once they are available in the literature. 

## Conclusions

The different thyroiditis variants are treated medically, and surgery is rarely indicated. Treating surgeons should consider all the factors affecting their decisions to operate on this special group of patients, including indications for surgery, improvement in local symptoms post-thyroidectomy, risk of thyroid cancer, intraoperative difficulty, and postoperative complications. Thus, each case should be individualized in the decision to operate or not by assessing the benefit-risk ratio. Future reviews of surgery for thyroiditis cases are suggested to include more studies designed as systematic reviews and meta-analyses, randomized controlled studies, and prospective cohort studies once they are available in the literature. 
